# Patients with stage IV epithelial ovarian cancer: understanding the determinants of survival

**DOI:** 10.1186/s12967-020-02295-y

**Published:** 2020-03-23

**Authors:** Yohann Dabi, Cyrille Huchon, Lobna Ouldamer, Sofiane Bendifallah, Pierre Collinet, Alexandre Bricou, Emile Daraï, Marcos Ballester, Vincent Lavoue, Bassam Haddad, Cyril Touboul, For the Groupe de Recherche FRANCOGYN

**Affiliations:** 1 Department of Obstetrics and Gynecology, Centre Hospitalier Intercommunal, Faculté de médecine de Créteil UPEC-Paris XII, Créteil, France; 2 Department of Gynecology and Obstetrics, Intercommunal Hospital Centre of Poissy – Saint Germain – en – Laye, 78103 Poissy, France; 3 Department of Obstetrics and Gynecology, Centre hospitalier régional universitaire de Tours, hôpital Bretonneau, Tours, France; 4 Department of Gynecology and Obstetrics, Tenon University Hospital, Assistance Publique, Hôpitaux de Paris (AP-HP) des, University Pierre and Marie Curie, Paris 6, Institut Universitaire de Cancérologie (IUC), Paris, France; 5 Department of Obstetrics and Gynecology, Centre Hospitalier Régional Universitaire, Lille, France; 6 Department of Obstetrics and Gynecology, Jean-Verdier University Hospital, Assistance Publique, Hôpitaux de Paris (AP-HP) des, Paris, France; 7 CRLCC Eugène-Marquis, Department of Gynecology, CHU de Rennes, Université de Rennes 1, Rennes, France; 8 Service de Gynécologie Obstétrique, Hôpital Intercommunal de Créteil, 40 Avenue de Verdun, Créteil, 94000 France

**Keywords:** Ovarian cancer, Stage IV, Debulking surgery, Chemotherapy, Prognostic factors, Post-operative residual disease

## Abstract

**Background:**

The most appropriate management for patients with stage IV ovarian cancer remains unclear. Our objective was to understand the main determinants associated with survival and to discuss best surgical management.

**Methods:**

Data of 1038 patients with confirmed ovarian cancer treated between 1996 and 2016 were extracted from maintained databases of 7 French referral gynecologic oncology institutions. Patients with stage IV diseases were selected for further analysis. The Kaplan–Meier method was used to estimate the survival distribution. A Cox proportional hazards model including all the parameters statistically significant in univariable analysis, was used to account for the influence of multiple variables.

**Results:**

Two hundred and eight patients met our inclusion criteria: 65 (31.3%) never underwent debulking surgery, 52 (25%) underwent primary debulking surgery (PDS) and 91 (43.8%) neoadjuvant chemotherapy and interval debulking surgery (NACT-IDS). Patients not operated had a significantly worse overall survival than patients that underwent PDS or NACT–IDS (p < 0.001). In multivariable analysis, three factors were independent predictors of survival: upfront surgery (HR 0.32 95% CI 0.14–0.71, p = 0.005), postoperative residual disease = 0 (HR 0.37 95% CI 0.18–0.75, p = 0.006) and association of Carboplatin and Paclitaxel regimen (HR 0.45 95% CI 0.25–0.80, p = 0.007).

**Conclusions:**

Presence of distant metastases should not refrain surgeons from performing radical procedures, whenever the patient is able to tolerate. Maximal surgical efforts should be done to minimize residual disease as it is the main determinant of survival.

## Background

Ovarian cancer remains the leading cause of death from gynecological cancer in developed countries [1]. The lack of effective screening results in a majority of patients diagnosed with advanced stage diseases and around 20% of the newly diagnosed patients have a stage IV disease [2]. Standard of care for patients with epithelial ovarian carcinoma (EOC) include the combination of a cytoreductive surgery to achieve no residual disease and a platinum–based chemotherapy regimen [3, 4].

The most important and independent prognostic factor identified in EOC is the amount of residual disease following cytoreductive surgery. The largest the residual tumor, the worst the prognosis is [3, 5, 6]. To minimize residual disease following surgery, radical and even ultra–radical surgery must be performed sometimes. Radical surgery comprising, in addition to the hysterectomy and bilateral adnexectomy, total omentectomy and appendicectomy +/− pelvic and para aortic lymphdenectomy, en bloc removal of the uterus, both ovaries, the pelvic peritoneum and recto-sigmoid with or without simple peritonectomies. Ultra-radical surgery, that is, a radical procedure plus at least one of the following: extensive peritonectomies including partial resection of the diaphragm or resection of subcapsular liver metastases, cholecystectomy, or splenectomy, resection of that tail of the pancreas and/or other bowel resection, partial gastrectomy, etc. [7]. The surgery is all the more aggressive that the disease is extensive and by definition, patients with stage IV disease have the most extensive disease [8, 9].

The cost to achieve a complete resection is a high perioperative morbidity and mortality. Indeed, complications in as high as 50% of cases have been reported when treating patients with EOC by radical surgery [10, 11]. Patients not eligible to primary cytoreductive surgery undergo neoadjuvant chemotherapy followed by interval debulking surgery. While neoadjuvant chemotherapy did reduce surgical complexity and increased the number of patients with complete resection, it is at best non inferior to upfront surgery for survival [4, 12, 13].

Unfortunately, there is only few data regarding specifically patients with stage IV disease [14–16] as many reports usually pool analysis with patients staged IIIC. These patients have the most extensive intra-abdominal disease associated with distant metastases and complete removal of disease requires radical procedures and possibly extra-abdominal surgery. Such procedures are highly morbid and could delay important adjuvant chemotherapy [17, 18]. Should these patients undergo highly morbid radical/ultra–radical procedures if surgery does not better than chemotherapy only? Is postoperative residual disease also an important prognostic factor in this specific subgroup of patients? Few authors addressed the question of their management and it remains unclear whether they do benefit from any surgery at all and if yes, if the most appropriate management is primary debulking surgery or neoadjuvant chemotherapy followed by interval debulking surgery [19–21]. Eventually, the understanding of the determinants of survival in this population is poor.

Our study therefore focused on understanding the main predictors of survival in patients with stage IV EOC. Our secondary objective was to discuss the most appropriate management in this specific population with extensive peritoneal disease and distant metastases.

## Methods

We conducted a retrospective study using maintained databases from 7 French referral gynecologic oncology institutions (University departments of gynecology of Creteil, Tenon, Poissy, Lille, Tours, Bondy and Rennes Hospitals). These databases registered all patients diagnosed with ovarian cancer at any stage between January 1996 and December 2016. The research protocol was approved by the Institutional Review Board (IRB) of the French College of Obstetrics and Gynecology (CEROG 2016-GYN-1003).

Patients with stage IV EOC were then selected for further analysis. As the International Federation of Gynecology and Obstetrics (FIGO) classification for EOC changed over the years, all patients’ stages were reassessed using the latest version of the classification and stage IV was defined as the presence of any distant metastasis, including inguinal lymph node metastasis and pleural effusion [22]. Patients with pleural effusion had a cytology by fine needle aspiration to confirm the presence of malignant cells (stage IVA). Exclusion criteria were: (i) Patients with non-epithelial tumors; (ii) Patients with undetermined FIGO stage; (iii) Patients with numerous missing data; (iv) Patients that underwent explorative and/or cytoreductive surgery elsewhere.

Furthermore, we excluded from analysis patients never operated because of poor general condition as well as those that refused surgical management.

All patients underwent clinical examination. Performance status was evaluated at the time of initial management to evaluate whether the patient could bear surgery and/or chemotherapy. Patients presenting with old age were assessed by the oncogeriatric.

Preoperative workup included at least an abdominal–pelvic computed tomography scan (CT–scan) and most patients had a blood test to assess Carbohydrate Antigen 125 (CA125) level. Each patient’s case was systematically reviewed by a multidisciplinary board that included at least a certified an oncologist, a pathologist, a radiologist and an experimented surgeon. All surgeries were performed by a qualified gynecologic oncologist, trained to digestive resections and to supra—mesocolic surgery. Initial management consisted in an explorative laparoscopy to determine the extent of the peritoneal spread and the resectability of the disease. Decision to proceed to either primary debulking surgery (PDS) or neoadjuvant chemotherapy then interval debulking surgery (NACT—IDS) was then decided based on the resectability, if the patient could bear it as recommended. Cytoreductive surgeries were always performed with intend to achieve no residual disease. It included at least a midline laparotomy, total hysterectomy, bilateral salpingo–oophorectomy and infragastric omentectomy. A more extensive surgery was performed when indicated and could involve digestive tract resections, upper abdominal resections (UAR) such as diaphragmatic resection, splenectomy, lymph nodes dissections, hepatectomy, and any other gesture to obtain no residual disease. Surgical complexity was assessed using the complexity score described by Aletti et al. [23].

All patients received chemotherapy regimens that were platinum based. Chemotherapy was started usually between 4 and 6 weeks following surgery but in cases of postoperative complications, this delay could be longer (until 2 month). Patients not fitting for PDS were surgically reevaluated after 2 to 4 cycles of NACT, depending on the centers. Patients non resectable following 6 cycles of NACT were considered never resectable and were treated with chemotherapy alone, if they could tolerate it. Second line chemotherapy for patients that progressed during NACT was decided with the referent oncologist.

Post-operative residual disease (RD) after PDS or IDS were classified as defined by Chang and Bristow [24]: no RD when all disease was removed, optimal when the largest tumor left was between 0.1 and 1 cm^2^ and gross RD when it was > 1 cm^2^.

### Postoperative management

All patients had a post-operative visit at 1 month. Post-operative complications were classified using the validated Clavien—Dindo scale [25]. Severe post-operative complications were those graded ≥ 3. Regular follow up consisted in clinical examination and blood testing including CA 125 dosage. Abdominal pelvic CT–scan was performed either if there was a clinical suspicion of recurrence or at least every 4 months for 2 years and every 6 months after. Disease recurrence was diagnosed on biopsy or imaging exam. Date of initial explorative surgery was used to calculate Overall Survival (OS) as well as the Progression Free Survival (PFS).

### Statistical analysis

Databases were managed using Excel (Microsoft Corporation, Redmond, WA, USA) and statistical analyses were performed using R software (3.3.1 version, available online). Statistical analysis was based on the Student’s t test for continuous variable and the χ^2^ test or Fisher’s exact test for categorical variables. The Kaplan–Meier method was used to estimate the survival distribution. Comparisons of survival were made using the log rank test. A Cox proportional hazards model including all the parameters statistically significant in univariate analysis, was used to account for the influence of multiple variables. Values of p < 0.05 were considered to denote significant differences (Additional file 1: Fig. 1).

## Results

### Main characteristics of the patients included

**Fig. 1:**
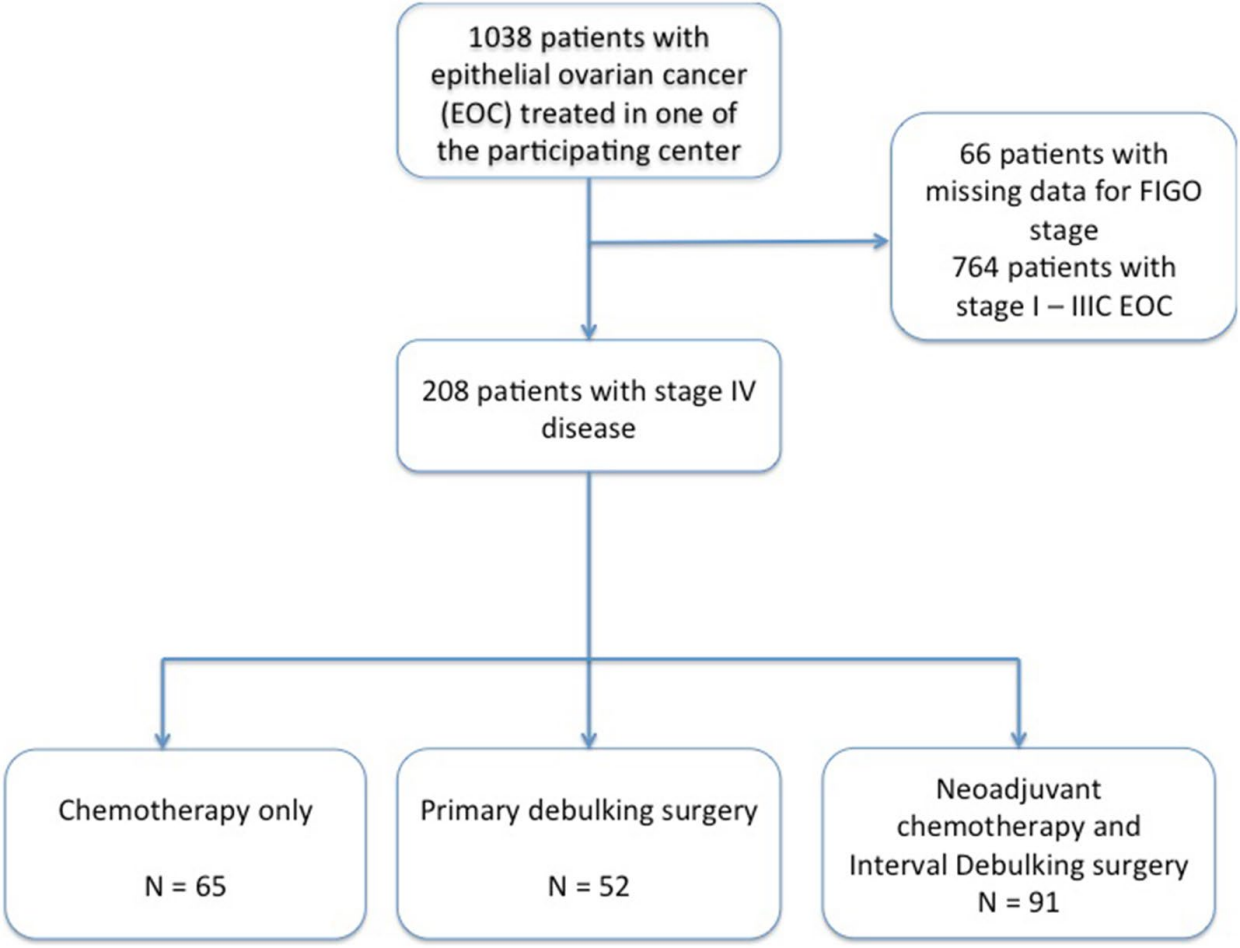
Flow chart of the study

Between 1996 and 2016, 1038 patients were treated for an ovarian cancer within our institutions. Of these, 208 met our inclusion criteria: 65 had chemotherapy alone, 52 underwent PDS and 91 NACT-IDS (Fig. 1).

Sixteen patients (7.7%) with stage IV disease were of poor general condition following chemotherapy not suitable for surgery and 4 patients refused surgical management. The other patients treated with chemotherapy alone were so because of disease progression during chemotherapy.

The main characteristics of the patients included are displayed in Table 1. Patients treated solely with chemotherapy were significantly older than the others (p = 0.009). Patients treated with up–front surgery had significantly less often serous histological subtype (p < 0.001) and lower CA125 level (p = 0.04). Patterns of metastases locations were similar between the groups except for a lower rate of pleural effusion within patients treated with up–front surgery (p = 0.03).

**Table 1:** Main characteristics of the patients included

	Chemotherapy only N = 65	Primary debulking Surgery N = 52	Interval debulking surgery N = 91	p-value
Age (years)	68.4 (57–78)	56.6 (47.8–67.25)	58.8 (51–66.5)	*< 0.001*
BMI	25.0 (21.0–27.5)	26.7 (22.1–29.7)	24.6 (20.4–26.4)	0.16
Histology				
Serous	57 (87.7)	30 (57.7)	73 (80.2)	*< 0.001*
Mucinous	1 (1.5)	2 (3.8)	1 (1.1)	
Endometrioid	0	4 (7.7)	9 (9.9)	
Transitional cell	0	1 (1.9)	0	
Clear cell	1 (1.5)	4 (7.7)	0	
Mixed	0	6 (11.5)	5 (5.5)	
Other	3 (4.6)	3 (5.8)	2 (2.2)	
Unknown	3 (4.6)	2 (3.8)	1 (1.1)	
Metastasis site				
Intrahepatic	22 (33.8)	14 (26.9)	20 (22.0)	0.25
Intra abdominal organ metastases^a^	11 (16.9)	16 (30.8)	15 (16.5)	0.3
Pleural effusion	23 (35.4)	9 (17.3)	41 (45.1)	*0.003*
Pleural metastasis	8 (12.3)	3 (5.8)	11 (12.1)	0.42
Over diaphragmatic metastases	16 (24.6)	12 (23.1)	24 (26.4)	0.9
Unknown	16 (24.6)	0	0	
CA125 level at diagnosis	4450 (378–2275)	1451 (227–1553)	2499 (530–3193)	*0.04*
Initial chemotherapy				
Received platinum	44 (67.7)	47 (90.4)	78 (85.7)	*0.002*
Received taxane	40 (61.5)	40 (76.9)	73 (80.2)	*0.03*

Data are given as mean (interquartile range) or n (%)

^a^Intra abdominal organs metastases: pancreas, spleen, digestive mucosa

The complexity of the surgery was higher for the patients treated with PDS when compared to those treated with NACT IDS (23/52, 44.2% vs 24/91, 26.4% for highly complex procedures, p = 0.01). There were a significantly higher proportion of patients with no RD in the group treated by PDS (40/52 76.9% vs 49/91 53.8%, p = 0.01) (Table 2). The intraoperative complications in the group of patients treated with primary debulking surgery were as follow: 1 nervous lesion, 1 urinary, 3 hemorrhages, 1 pneumothorax. The complications in the group of patients treated with neoadjuvant chemotherapy and interval debulking surgery were as follow: 5 vascular lesions (hemorrhages), 7 pneumothorax, 1 digestive.

**Table 2:** Surgery outcomes in patients with stage IV ovarian cancer

	Primary debulking surgery N = 52	Neo-adjuvant chemotherapy–interval debulking surgery N = 91	p-value
Bowel resection	24 (46.1)	29 (31.9)	0.12
Upper abdominal surgical procedures^a^	30 (57.7)	37 (40.7)	0.07
Complexity of the surgery			*0.01*
Low	15 (28.8)	19 (20.9)	
Intermediate	14 (26.9)	48 (52.7)	
High	23 (44.2)	24 (26.4)	
Intraoperative complication	6 (11.5)	13 (14.3)	0.83
Postoperative residue			
Optimal cytoreduction RD < 1 cm^b^	3 (5.8)	2 (2.2)	0.3
Complete resection: no RD	40 (76.9)	49 (53.8)	*0.01*
Postoperative complications	25 (48.1)	28 (30.8)	0.06

Data are given as n (%)

*RD* residual disease, * NA* not applicable

^a^ Upper abdominal surgical procedures = splenectomy, gallbladder resection, liver resection, diaphragmatic resection

^b^ Largest residual tumor nodule measuring 1 cm or less

Twenty-five (48.1%) and 28 (30.8%) patients experienced post-operative complications in the PDS and NACT–IDS groups, respectively. The complications in the group of patients treated with primary debulking surgery were as follow: 5 infections, 6 embolism, 4 respiratory, 8 digestive, 2 bleeding. The complications in the group of patients treated with neoadjuvant chemotherapy and interval debulking surgery were as follow: 5 infections, 7 respiratory, 6 digestive, 4 bleeding, 3 lymphocele, 3 others.

There was a trend toward more complications in patients treated with PDS without reaching statistical significantly (p = 0.06). None of the patients operated died within 30 days (immediate post-operative death).

### Survival analysis

Median follow up was of 19.5 months (min: 0.2 max: 130.6).

**Fig. 2:**
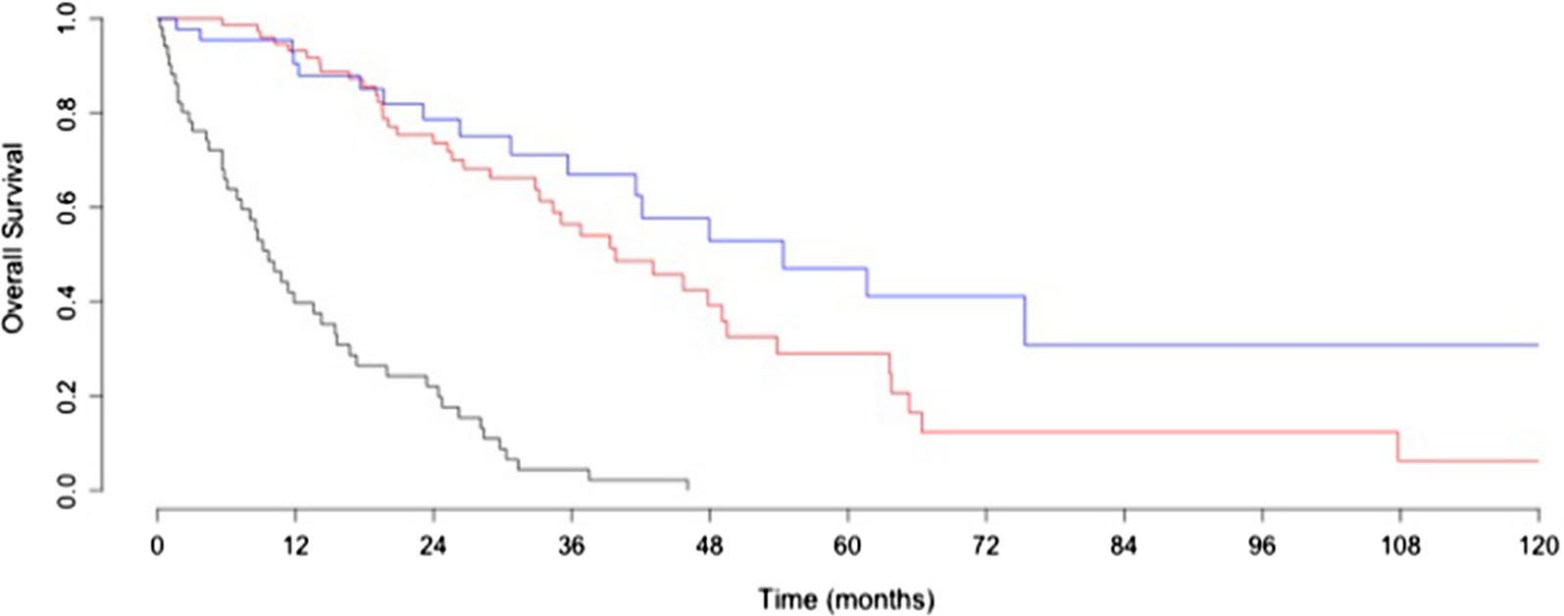
Kaplan Meier curve for overall survival stratified by initial management. In red dashed line: patients with surgical staging. In black: Patients treated with chemotherapy only; In Red: patients treated by NACT–IDS; In blue: patients treated by PDS. Patients not operated had a significantly worse prognostic than patients operated (p < 0.001)

Patients treated solely with chemotherapy had a significantly worse progression free survival (p < 0.001) and overall survival than patients that underwent PDS or NACT–IDS (p < 0.001) (Fig. 2 and Additional file 1: Fig. 1). Patients treated with up front surgery had longer progression free and overall survival than those treated with NACT–IDS (p < 0.001 and p = 0.03, respectively).

**Fig. 3:**
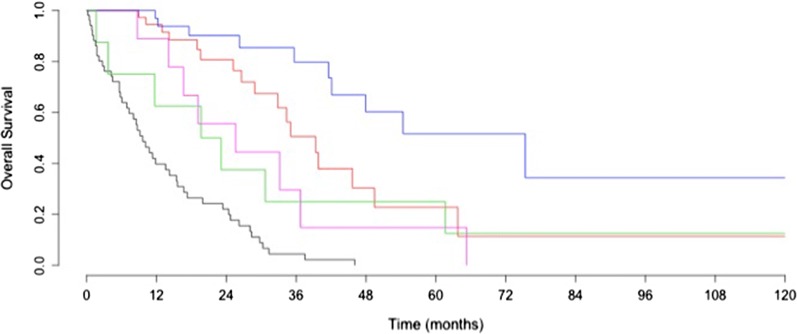
Kaplan Meier curve for overall survival in patients operated stratified by postoperative residual disease. In black: Patients with chemotherapy only; In blue and green: patients treated by PDS with no residual disease and with macroscopic residual disease, respectively; In red and purple: patients treated by PDS with no residual disease and with macroscopic residual disease, respectively. Survival was significantly better in patients with no RD following surgery, whichever initial management (PDS or NACT–IDS) (p < 0.01)

Kaplan–Meier curves for overall survival in patients operated stratified by post-operative RD are presented in Fig. 3. Patients with no RD following surgery (PDS or NACT–IDS) and those with non–null postoperative RD had similar PFS but the latest had worse overall survival (p < 0.01).

### Cox model on factor influencing overall survival

In univariate analysis, an age > 65 years at the time of diagnostic (p < 0.001), the surgery act (upfront surgery or after neoadjuvant chemotherapy) (p < 0.001), post-operative residual disease (p < 0.001), a platinum and taxol based chemotherapy (p < 0.001) and the presence of intra-abdominal organ metastases (p = 0.0209) were all significantly associated with overall survival (Table 3).

**Table 3:** Cox models

Variables	Univariable analysis		Multivariable analysis	
	HR (95% CI)	p-value	HR (95% CI)	p-value
Age > 65 years	2.4 (1.6–3.6)	< 0.001		
Surgical complexity		0.104	–	–
Low	Ref			
Intermediate	1.2 (0.7–2.3)			
High	0.5 (0.3–1.0)			
CA 125 > 500	1.0 (0.6–1.6)	0.96	–	**–**
Chemotherapy only	Ref	< 0.001		
NACT– IDS	0.2 (0.1–0.3)		0.48 (0.22–1.01)	*0.054*
PDS	0.1 (0.1–0.2)		0.32 (0.14–0.71)	*0.005*
Postoperative residual disease > 0	Ref	< 0.001		
Postoperative residual disease = 0 (complete surgery)	0.2 (0.20–0.21)		0.37 (0.18–0.75)	*0.006*
Association of Carboplatin and Paclitaxel	0.4 (0.2–0.56)	< 0.001	0.45 (0.25–0.80)	*0.007*
Metastatic locations				
Hepatic	1.3 (0.8–2.1)	0.213	–	–
Pleural effusion	1.5 (1.0–2.3)	0.0658	–	–
Solid pleural lesion	1.2 (0.6–2.6)	0.576	–	–
Supra-diaphragmatic lesion	1.1 (0.7–1.8)	0.693	–	–
Deep intra—abdominal organ	0.5 (0.2–0.95)	0.0209		

Univariable and multivariable analysis of the factors influencing overall survival in patients with stage IV epithelial ovarian cancer

In multivariable analysis, three factors were independent predictors of survival: upfront surgery (HR 0.32 95% CI 0.14–0.71, p = 0.005), post-operative residual disease = 0 (HR 0.37 95% CI 0.18–0.75, p = 0.006) and association of Carboplatin and Paclitaxel regimen (HR 0.45 95% CI 0.25–0.80, p = 0.007). The other parameters were not independent predictors of survival in our cohort (Table 3).

## Discussion

We found patients diagnosed with stage IV ovarian cancer had benefit from debulking surgery with improved PFS and OS when compared to non-operated patients.

Debulking strategy in ovarian cancer results from more than 40 years of surgical evolution and understanding of the disease. Postoperative residual disease is, to date, the best predictor of survival in patients with advanced disease [3, 5, 6]. However, the literature is unclear regarding the benefit patients with distant organ metastases could harvest from debulking surgery to complete resection of the disease and if they do, which of them are and how to select them. Some authors defend the idea stage IV of the disease should be considered as a distinct clinic-pathological entity, with specific aggressiveness. We feel the conclusions of the studies that included a majority of stage IIIC patients should not be extrapolated to patients with stage IV. The major result of our study is the predominance of intra-peritoneal disease on overall survival, despite significant rate of distant organ metastasis.

Perri et al. showed isolated thoracic recurrences, without concomitant abdominal recurrence, are anecdotic [26]. This highlights the importance of abdominal control of the disease in these patients. Patients treated with chemotherapy alone had worse prognostic than those operated, despite important complications rates (48.1% and 30.8% in patients operated that undergone PDS and NACT–IDS, respectively) and frequent upper abdominal procedures. As we were not able to report performans status in our cohort, it remains possible patients never operated were in worse condition prior to any treatment, with more comorbidities and more aggressive diseases, which could bias our results. However, only a limited number of patients were treated with chemotherapy alone because of poor general condition following chemotherapy. Furthermore, we found patients operated had not only improved overall survival but also progression free survival. Response to chemotherapy could be an interesting parameter to help clinicians selecting the patients that will benefit from radical/ultra–radical surgery [27].

We found primary debulking surgery was an independent predictor of survival. New studies keep fueling the debate between upfront surgery and neoadjuvant chemotherapy. Usually, neoadjuvant chemotherapy is preferred for patients with initially non–resectable disease. Makar et al. in their recent review of the literature concluded stage IV disease was not a contraindication to PDS but neoadjuvant chemotherapy is preferable in cases of multiple intrahepatic/lung metastases or with massive ascites with miliary spread [28]. The management of the patients in our series was in accordance with makar et al. conclusions, explaining why the populations weren’t totally comparable. A limitation of our work is that data regarding the peritoneal cancer index (PCI) is missing. Some upper abdominal areas disease are known to predict poor oncological outcomes [29]. Furthermore, the better survival in patients treated with upfront surgery could reflect a lower disease burden initially. Eventually, The fact that the surgical complexity was not associated with survival suggests that extended surgeries could have overtook the bad prognosis of extended intra-peritoneal diseases. This paradigm was also raised by other authors [30].

Postoperative residual disease was a strong independent predictor of survival in our cohort, which justifies surgical effort to achieve no abdominal residual disease. Other studies suggested this factor could be important, as summarized by Ataseven et al. in their recent review [31]. The fact that none of the metastases locations was associated with survival, along with the impact of residual disease following surgery, underline the prognosis of these patients is driven by the evolution of the abdominal disease. One limit of our study is that precise location of the metastases is not available, which could possibly impact survival. However, our results are in line with those reported by Winter et al. [32] showing better survival when complete surgery is achieved, despite a distant metastasis rate of 12.5% and hepatic metastasis of 17.8%. In their 360 patients’ cohort, survival significantly decreased from 5 cm^2^ of residual disease. It is of note that even those with postoperative residual disease between 0.1 and 5 cm benefited from the surgery. In our cohort, none of the patients had extra abdominal procedures performed before or during the cytoreductive surgery. Most of the literature focused on thoracic procedures, whose safety has been proven but not their impact on prognostic [33]. Bristow et al. reported increased survival in patients with intrahepatic metastases undergoing complete disease removal, both abdominal and hepatic [16].

The use of a combined platinum and taxane based chemotherapy regimen had an independent positive impact on overall survival in our cohort, further confirming the results reported by Atavensen et al. [31]. The importance of the chemotherapy is enhanced by the presence of extra–abdominal disease that cannot be cured otherwise. In the cohort of Winter, the patients had chemotherapy based on Cisplatin and Paclitaxel, which is not the standard regimen anymore since the results reported by Ozols [34], Trimbos [35] and Nejit [36]. A matter of debate in patients with advanced ovarian cancer (stage III–IV) was the possible delay to initiate chemotherapy following cytoreductive surgery especially in patients undergoing radical procedures. A recent meta-analysis by Mahner et al. [37] concluded a delayed chemotherapy was associated with early disease recurrence and significantly decreased OS following initial surgery in patients with complete resection. As so, inappropriate timing of chemotherapy could compromise the surgical effort to achieve no residual disease resulting in a double negative effect with high postoperative morbidity and shorten survival. Thus, it is imperative to select patients that will benefit from an upfront radical surgery and those eligible to NACT–IDS to attempt reducing postoperative morbidity. The use of nomograms could be promising in this purpose. These tools have been validated in other malignancies to predict at a patient level the probability of a certain event to occur. Shim et al. developed a model for predicting incomplete cytoreduction in advanced ovarian cancer with good performances [38]. Others focused on developing prediction models for immediate postoperative complications. Should they be validated, these nomograms could be used to help surgeons determine the most appropriate management for patients with advanced diseases. Finally, we cannot emphasize more the role of the surgeon’s experience and the institution to decide which management fits a certain patient best as it has been demonstrated before [39]. More studies are required to define better the indications of PDS and NACT IDS in stage IV advanced ovarian cancer.

## Conclusion

We demonstrated patients with stage IV ovarian cancer benefit from a cytoreductive surgery, with the most important prognostic factor being postoperative residual disease. Presence of distant metastases should not refrain surgeons from performing radical or ultra-radical procedures, whenever the patient is able to tolerate it. Extensive efforts should be done to minimize residual disease in these patients as their prognostic seems driven by the abdominal disease extension.

## Supplementary information

**Additional file 1: Fig.** **1.** Kaplan–Meier curve for progression free survival stratified by initial management. Red dashed line: patients with surgical staging. In black: Patients treated with chemotherapy only; In Red: patients treated by NACT – IDS; In blue: patients treated by PDS. Patients not operated had a significantly worse prognostic than patients operated (p < 0.001).

## Data Availability

Not applicable.
