# Supplementary figures and images for: Patients with stage IV epithelial ovarian cancer: understanding the determinants of survival
Source: J Transl Med. 2020 Mar 23;18:134. doi: 10.1186/s12967-020-02295-y (PMC7087387; doi:10.1186/s12967-020-02295-y)

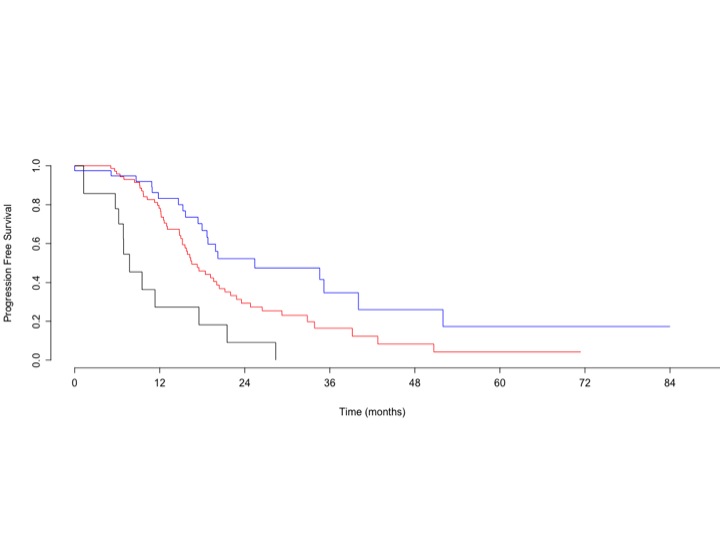

Supplement: Supplementary file 1 — Additional file 1: Fig. 1. Kaplan–Meier curve for progression free survival stratified by initial management. Red dashed line: patients with surgical staging. In black: Patients treated with chemotherapy only; In Red: patients treated by NACT – IDS; In blue: patients treated by PDS. Patients not operated had a significantly worse prognostic than patients operated (p < 0.001). [file 12967_2020_2295_MOESM1_ESM.jpg]
